# Ruthenium nanoclusters modified by zinc species towards enhanced electrochemical hydrogen evolution reaction

**DOI:** 10.3389/fchem.2023.1189450

**Published:** 2023-04-06

**Authors:** Hefeng Zhang, Shengliang Qi, Kaixin Zhu, Xu Zong

**Affiliations:** Marine Engineering College, Dalian Maritime University, Dalian, China

**Keywords:** hydrogen evolution reaction, water splitting, electrocatalysis, Ru nanoclusters, Zn species

## Abstract

Ruthenium (Ru) has been considered a promising electrocatalyst for electrochemical hydrogen evolution reaction (HER) while its performance is limited due to the problems of particle aggregation and competitive adsorption of the reaction intermediates. Herein, we reported the synthesis of a zinc (Zn) modified Ru nanocluster electrocatalyst anchored on multiwalled carbon nanotubes (Ru-Zn/MWCNTs). The Ru-Zn catalysts were found to be highly dispersed on the MWCNTs substrate. Moreover, the Ru-Zn/MWCNTs exhibited low overpotentials of 26 and 119 mV for achieving current intensities of 10 and 100 mA cm^−2^ under alkaline conditions, respectively, surpassing Ru/MWCNTs with the same Ru loading and the commercial 5 wt% Pt/C (47 and 270 mV). Moreover, the Ru-Zn/MWCNTs showed greatly enhanced stability compared to Ru/MWCNTs with no significant decay after 10,000 cycles of CV sweeps and long-term operation for 90 h. The incorporation of Zn species was found to modify the electronic structure of the Ru active species and thus modulate the adsorption energy of the H_ad_ and OH_ad_ intermediates, which could be the main reason for the enhanced HER performance. This study provides a strategy to develop efficient and stable electrocatalysts towards the clean energy conversion field.

## 1 Introduction

As one of the carbon-free energy sources, hydrogen (H_2_) has been recognized as a promising alternative to the non-renewable fossil fuels for its high energy density, zero emission and cleanness (Dresselhaus and Thomas, 2001; Turner, 2004). Compared to the traditional approach of producing H_2_ from reforming of fossil fuels, electrochemical water splitting has been widely accepted as a green and efficient method (Lee, et al., 2022). In this process, efficient electrocatalysts for the hydrogen evolution reaction (HER) are crucial, among which, platinum (Pt)-group materials have been regarded as the benchmark catalysts (Yusuf, et al., 2023).

As a member of Pt-group metals, ruthenium (Ru) has attracted great attention due to its high efficiency in water dissociation, moderate Ru-H bond, and relatively lower price compared to the Pt-based catalyst (Luo, et al., 2020; Wu, et al., 2021). However, Ru nanoparticles were prone to aggregate, leading to decreased activity and stability (Ma, et al., 2022; Guan, et al., 2023). Moreover, subnanometric Ru nanoclusters were found to be more efficient than Ru single atoms and larger Ru nanoparticles due to the upshifted d-band center that enabled a stronger ability of water dissociation (Hu, et al., 2022). Therefore, great efforts have been devoted to improving the HER activity of Ru-based catalysts by regulating the particle size. For example, different carbon materials like multiwalled carbon nanotubes (MWCNTs), graphene, carbon nanospheres, etc., have been used as the substrates to immobilize and modify the size of Ru-based catalysts for HER (Barman, et al., 2017; Cheng, et al., 2018; Li, et al., 2018; Kweon, et al., 2020; Zhang, et al., 2021; Gao, et al., 2022).

Moreover, one of the key factors for the low HER kinetics observed on Ru was considered to be the large energy barrier of the Volmer step (H_2_O+ e^−^ → H_ad_ + OH^−^, H_ad_: the adsorbed hydrogen atom), which can be divided into two processes of H_2_O ⇋ H_ad_ + OH_ad_ and OH_ad_ + e^−^ ⇋ OH^−^ (Zhang et al., 2022a). The strong Ru-H_ad_ and Ru-OH_ad_ bonds will impede the desorption process and lead to the poisoning of the active sites. Therefore, it is important to modulate the Ru-based catalysts to obtain appropriate adsorption energy of the reaction intermediates. Incorporating a second non-noble metal heteroatom such as Mo (Tu et al., 2020), W (Meng et al., 2021), Co (Su et al., 2017; Li et al., 2021; Su et al., 2021), Bi (Zhao et al., 2023), etc., to form multiple active sites has been identified to be one of the effective strategies to modulate the binding strength of the intermediates (Wu, et al., 2021; Zhang et al., 2022b; Li, et al., 2022; Zhao, et al., 2023). We anticipate that similar concept can be extended to the synthesis of Ru-based catalyst with promising HER performances.

Herein, we present the synthesis of a Ru-Zn/MWCNTs electrocatalyst by modifying Ru nanoclusters with Zn species. The Ru-Zn/MWCNTs electrocatalyst showed a favorable activity with low overpotentials of 26 and 119 mV at current intensities of 10 and 100 mA cm^−2^, respectively, outperforming the commercial Pt/C. Furthermore, the Ru-Zn/MWCNTs electrocatalyst exhibited an excellent stability with no obvious decay after 10,000 cycles of the durability test, which is much superior to the Ru/MWCNTs without Zn modification. The addition of Zn species to the Ru-based catalyst was supposed to regulate the adsorption energy of the intermediate H_ad_ and OH_ad_ species, therefore contributing to the enhancement of HER performance. This study provides guidance for the design of low-cost, highly efficient and ultra-stable electrocatalysts.

## 2 Experimental section

### 2.1 Chemicals

Ruthenium chloride (RuCl_3_) and commercial Pt/C (5 wt%) were obtained from Shanghai Macklin Biochemical Co., Ltd. Hydroxylated multiwalled carbon nanotubes (MWCNTs, 20–30 nm, >98%) were supplied by Chengdu Institute of Organic Chemistry, Chinese Academy of Sciences. Zinc chloride (ZnCl_2_) was acquired from Shanghai Aladdin Biochemical Technology Co., Ltd.

### 2.2 Preparation of Ru/MWCNTs and Ru-Zn/MWCNTs

Different amounts of RuCl_3_ solution (9.74 mg mL^−1^) were added to 20 mL deionized water and ethanol (v/v = 3: 1) solution with hydrated MWCNTs (MWCNT-OH, 500 mg). The mixture was sonicated for 30 min, and then vigorously stirred at ambient temperature for 12 h. After drying in an oven at 60°C, the resultant materials were further annealed at different temperature (typically 600°C) in a 5%H_2_/Ar for 1 h. For the synthesis of Ru-Zn/MWCNTs, all the processes were the same, except that different amounts of ZnCl_2_ were added as the source of Zn species.

### 2.3 Characterizations

The transmission electron microscope (TEM) images of the catalysts were conducted using JEM-2100 with an accelerating voltage of 200 kV. X-ray powder diffraction (XRD) was carried out on a DH-2700 BH, Rigaku with Cu *K*α radiation (*λ* = 1.5418 Å) with 2θ ranging from 10 to 60°. The elemental compositions were determined using an inductively coupled plasma atomic emission spectrometer (ICP-AES, Agilent 725 ES). X-ray photoelectron spectroscopy (XPS) analysis was performed by a ThermoFischer ESCALAB 250Xi apparatus equipped with an Axis Supra spectrometer with a monochromatic Al Kα source operating at 15 mA and 14 kV.

### 2.4 Electrochemical measurements

The catalyst (5 mg) was dispersed in 1 mL of a mixed solution of ethanol (960 μL) and 5% Nafion (40 μL). The resulting mixture was sonicated for 30 min in an ice bath to form a uniform catalyst ink.

Electrochemical measurements were carried out in a conventional three-electrode system on a CHI660E Electrochemical Workstation (Shanghai Chenhua Instrument Corporation, China). A graphite rod was used as the counter electrode, and Hg/HgO electrode was taken as the reference electrode. A glassy carbon electrode (GCE, diameter: 3 mm, area: 0.071 cm^2^) was used as the working electrode to support the catalyst ink (25 μg) for electrochemical tests. In a 1 M KOH solution, linear sweep voltammetry (LSV) was used to evaluate the HER performances of the catalysts at a sweep rate of 5 mV s^−1^. All polarization curves were corrected for 90% iR. Electrochemical impedance spectroscopy (EIS) measurements were carried out at a frequency range of 0.1 Hz–100 kHz in a 1.0 M KOH solution. The durability test was performed using chronoamperometry in 1.0 M KOH solution. In addition, the LSV curves after the 10,000th cycle of cyclic voltammetry (CV) sweeps were measured to further evaluate the stability of the catalysts.

## 3 Results and discussion

### 3.1 Synthesis of Ru-Zn/MWCNTs catalysts

**SCHEME 1:**
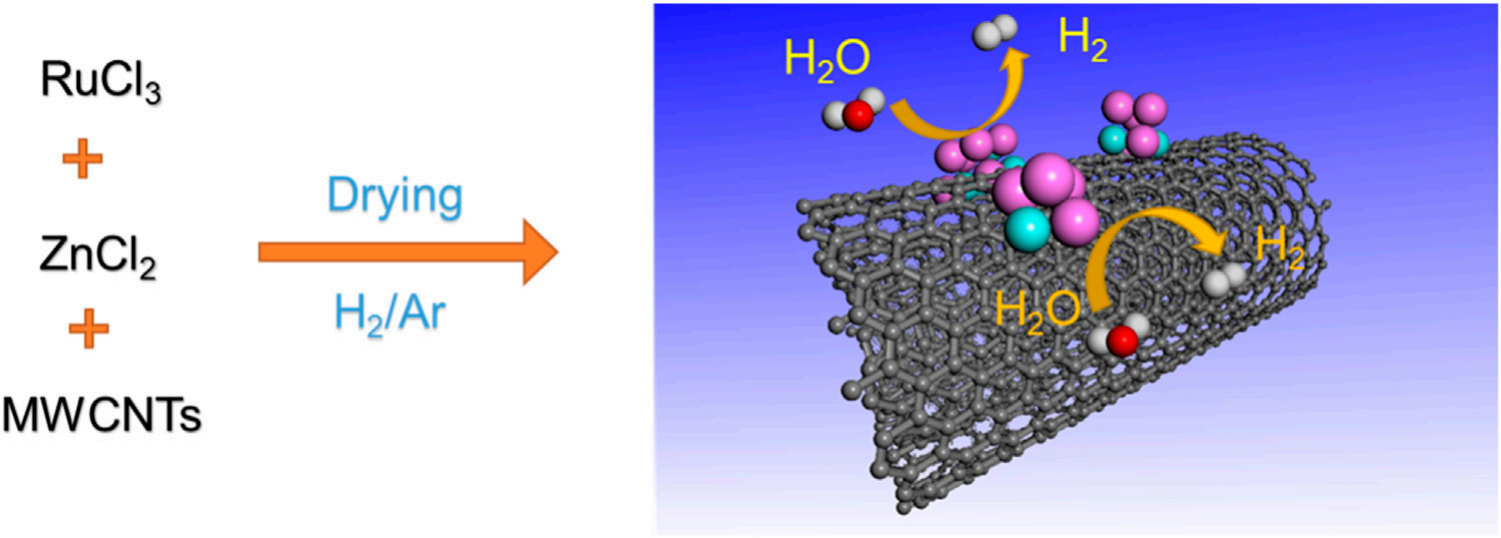
The synthesis process of Ru-Zn/MWCNTs.

A series of Ru-based catalysts were synthesized by anchoring different amounts of Ru^3+^ and Zn^2+^ ions on hydroxylated MWCNTs *via* a simple impregnation method, followed by H_2_/Ar reduction at 600°C for 1 h (Scheme 1). The obtained samples were denoted as *x* wt% Ru-Zn/MWCNTs, where *x* represented the total loading amounts of Ru and Zn species. Generally, the Ru-Zn/MWCNTs refers to samples with a Ru:Zn molar ratio of 1:1 if not otherwise stated.

### 3.2 XRD analysis of different Ru-Zn/MWCNTs electrocatalysts

**FIGURE 1:**
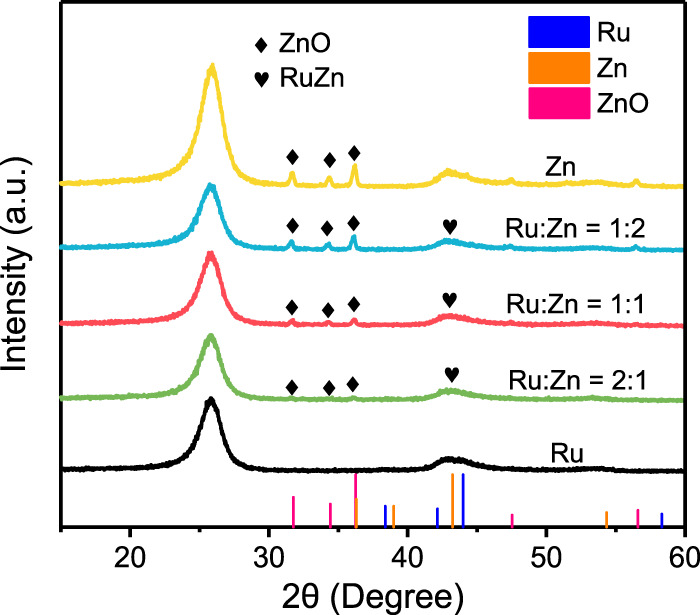
XRD patterns of the as-prepared 4 wt% Ru-Zn/MWCNTs samples at different Ru: Zn molar ratios, 4 wt% Ru/MWCNTs, and 4 wt% Zn/MWCNTs.

XRD were utilized to characterize the crystal structures of the different Ru-Zn/MWCNTs electrocatalysts, as shown in Figure 1. The 4 wt% Ru/MWCNTs sample featured the main peak at 2θ of 44.0°, representing the formation of metallic Ru (PDF: No. 06-0663) on MWCNTs. For the 4 wt% Zn/MWCNTs sample, three peaks at 31.8°, 34.4° and 36.3° were attributed to ZnO (PDF: No. 36-1451) and one peak at 43.2° originated from metallic Zn (PDF: No.04-0831). When both Ru and Zn were used to prepare the 4 wt% Ru-Zn/MWCNTs sample, peaks corresponding to both ZnO and the metallic phases were observed. With increasing the content of Zn species in 4 wt% Ru-Zn/MWCNTs, the peak intensity of ZnO increased, indicating an increase in the amount of ZnO species. Furthermore, no obvious splitting peaks appeared in the metallic phase, demonstrating the possibility of the formation of RuZn alloy. Therefore, Ru and Zn species in metallic state and ZnO were present in the 4 wt% Ru-Zn/MWCNTs samples.

### 3.3 HER activity of different Ru-Zn/MWCNTs samples

**FIGURE 2:**
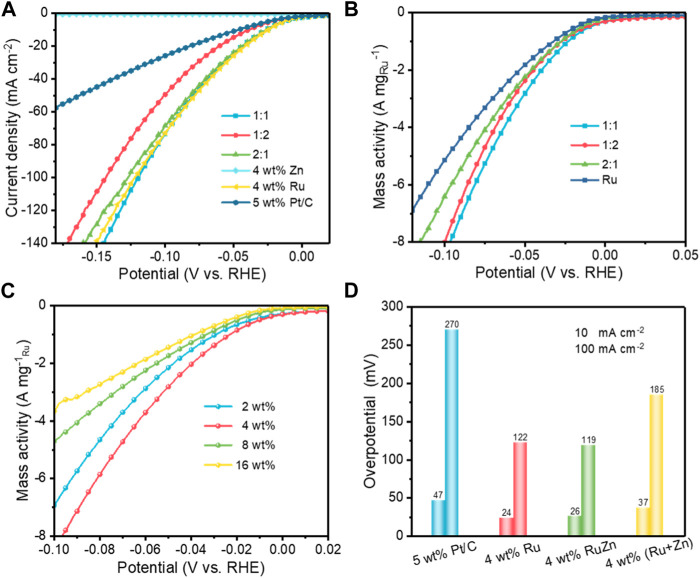
The electrocatalytic HER performances of the different Ru-based catalysts in 1 M KOH. **(A)** Polarization curves of 4 wt% Ru-Zn/MWCNTs catalysts at different Ru:Zn molar ratios with 90% iR correction; **(B)** The mass activity normalized by the amounts of Ru in the 4 wt% Ru-Zn/MWCNTs catalysts at different Ru:Zn molar ratios; **(C)** The mass activity of the *x* wt% Ru-Zn/MWCNTs catalysts with different total loadings of Ru and Zn at a Ru: Zn molar ratio of 1:1; **(D)** Overpotential comparison of the different Ru- and Zn-based catalysts at the current intensities of 10 and 100 mA cm^−2^.

The electrochemical HER performances of the as-prepared Ru-Zn/MWCNTs catalysts were evaluated by the linear sweep voltammetry (LSV) in a typical three-electrode electrochemical cell in 1.0 M KOH electrolyte (Figure 2; Supplementary Figure S1; Supplementary Table S1). The effects of the Ru:Zn molar ratio on the HER activities of the 4 wt% Ru-Zn/MWCNTs samples were first investigated. As shown in Figure 2A, 4 wt% Ru/MWCNTs displayed an overpotential of 24 mV and 122 mV for achieving current intensities of 10 and 100 mA cm^−2^, respectively, and the 4 wt% Zn/MWCNTs catalysts exhibited negligible HER activity. However, by incorporating Zn to the Ru/MWCNTs catalysts, a drastic increase in the HER activity was observed. Among all the 4 wt% Ru-Zn/MWCNTs, samples that with Ru:Zn molar ratio of 1:1 exhibited the best activity with overpotentials of 26 and 119 mV at current intensities of 10 and 100 mA cm^−2^, respectively. Moreover, all the 4 wt% Ru-Zn/MWCNTs catalysts exhibited even higher HER activity than the commercial 5 wt% Pt/C. By normalizing the content of Ru, the mass activity of 4 wt% Ru-Zn/MWCNTs was superior to that of the 4 wt% Ru/MWCNTs (Figure 2B) and the overall HER activity of 4 wt% Ru-Zn/MWCNTs at a molar ratio of 1:1 for Ru:Zn was comparable to that of the 4 wt% Ru/MWCNTs. Therefore, the incorporation of the Zn species can reduce the usage of Ru metal without sacrificing the HER performance.

In addition, we also evaluated the HER performances of the Ru-Zn/MWCNTs samples with different total metal loadings ranging from 2 wt% to 16 wt%. It was found that the HER activity of Ru-Zn/MWCNTs increased with the increase of the total metal loadings (Supplementary Figure S2), where the overpotentials were only 16 and 82 mV at current intensities of 10 and 100 mA cm^−2^, respectively, for the 16 wt% Ru-Zn/MWCNTs. This can be explained by the increase in the number of active sites. After normalization by the amount of Ru, the 4 wt% Ru-Zn/MWCNTs was found to exhibit the best HER performance (Figure 2C). Moreover, the mass activity of 4 wt% Ru-Zn/MWCNTs at −10 mV was 490.2 mA mg^−1^_Ru_, about 3.3 times higher than that of the commercial 5 wt% Pt/C (147.9 mA mg^−1^_Pt_), implying the superiority of the as-prepared Ru-Zn/MWCNTs electrocatalyst.

We also performed additional control experiments under the same conditions to investigate the role of Zn species in modifying the Ru catalysts. 4 wt% Ru/MWCNTs and 4 wt% Zn/MWCNTs were mechanically mixed with a molar ratio of 1:1. Overpotentials of 37 and 185 mV were required in the resulting mixed catalysts for achieving current intensities of 10 and 100 mA cm^−2^, respectively, which were higher than that required for the 4 wt% Ru-Zn/MWCNTs catalyst (Figure 2D). Therefore, it was reasonable to speculate that strong interactions existed between the Ru and Zn species for the Ru-Zn/MWCNTs catalyst while not for the mixed Ru/MWCNTs and Zn/MWCNTs electrocatalyst. The strong interaction between Ru and Zn will modify the electronic structures of the active Ru species and therefore lead to enhanced HER activity. Considering its highest mass activity, the 4 wt% Ru-Zn/MWCNTs at a Ru:Zn molar ratio of 1:1 was mainly investigated in the following studies (referred as Ru-Zn/MWCNTs).

### 3.4 Reaction kinetics analysis of the 4 wt% Ru-Zn/MWCNTs electrocatalyst

**FIGURE 3:**
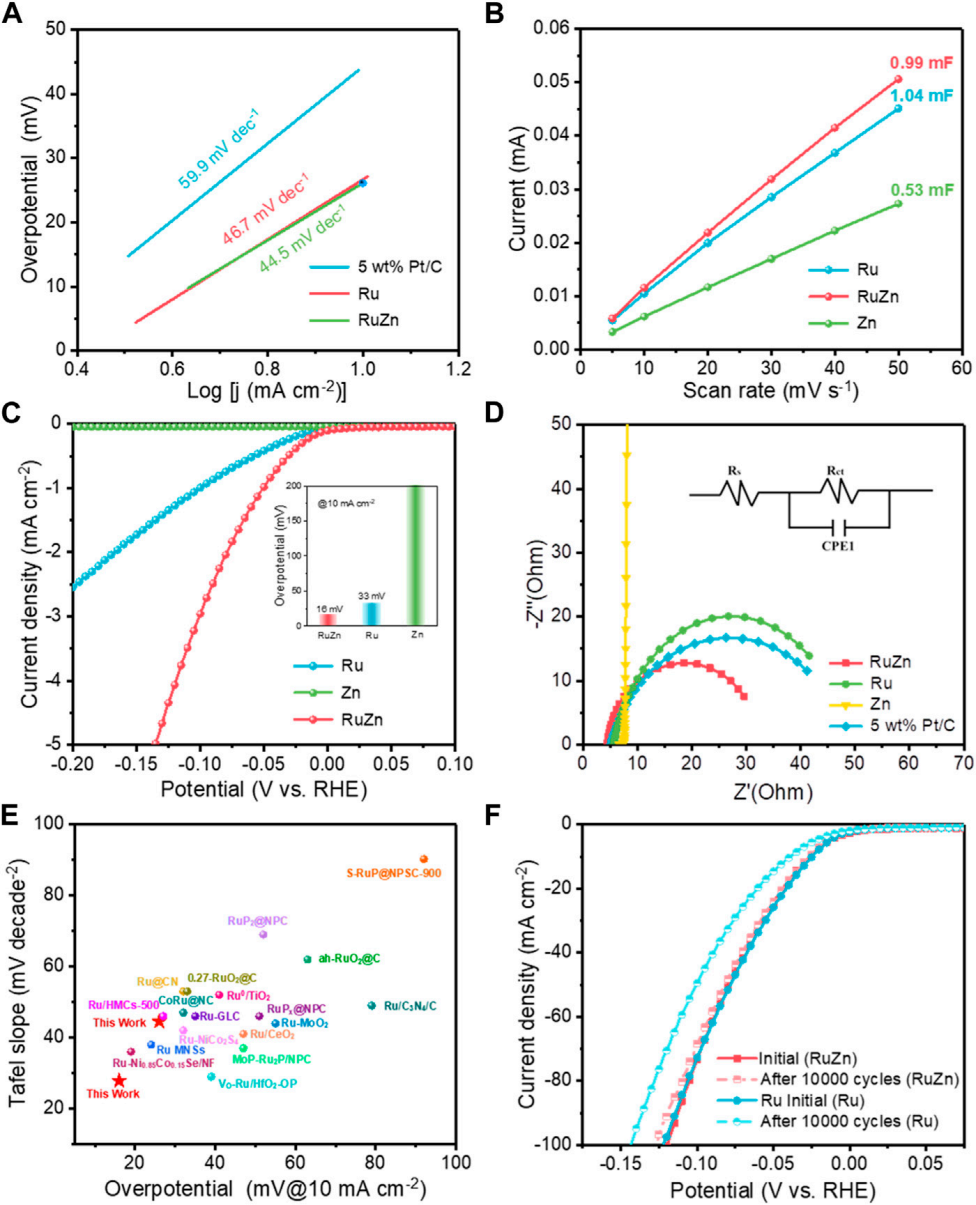
The electrocatalytic HER performances of the 4 wt% Ru-Zn/MWCNTs catalysts and the counterparts in 1 M KOH. **(A)** Tafel plots; **(B)** Capacitive currents against the scan rate and the corresponding C_dl_ values estimated through linear fitting of the plots; **(C)** The specific activity normalized by ECSA; **(D)** EIS Nyquist plots at overpotential of 50 mV and the corresponding equivalent circuit diagram; **(E)** Performances comparison of the Tafel slope and overpotential at a current intensity of 10 mA cm^−2^ with the Ru-based catalysts reported previously; **(F)** Duration test after 10,000 cycles of CV sweeps. (Ru, Zn and RuZn refer to 4 wt% Ru/MWCNTs, 4 wt% Zn/MWCNTs, and 4 wt% Ru-Zn/MWCNTs, respectively).

Next, Tafel slopes were further obtained from the Tafel plots to investigate the reaction kinetics. 4 wt% Ru-Zn/MWCNTs showed a low Tafel slope of 44.5 mV dec^−1^, which was smaller than that of 4 wt% Ru/MWCNTs (46.7 mV dec^−1^) and commercial 5 wt% Pt/C (59.9 mV dec^−1^) (Figure 3A). This clearly indicated a more favorable HER kinetics for Ru-based catalyst after incorporating Zn species. Moreover, compared with the 4 wt% Ru/MWCNTs and 4 wt% Zn/MWCNTs, the electrochemical double layer capacitance (C_dl_) of the 4 wt% Ru-Zn/MWCNTs were significantly increased (Figure 3B), indicating that the addition of Zn species can increase the electrochemical active surface area (ECSA) and the catalytic active sites of the Ru-based catalyst (Supplementary Figure S3). The ECSA can be obtained from C_dl_ normalized by specific capacitance (Wei, et al., 2019). Then, the LSV curves were further normalized to exclude the ECSA contribution and obtain the specific activity (Figure 3C), which showed that the 4 wt% Ru-Zn/MWCNTs catalyst exhibited a much higher HER performance than that of the 4 wt% Ru/MWCNTs. This revealed that the intrinsic activity of the Ru-based catalyst was remarkably improved by Zn species modification. Furthermore, the electrochemical impedance spectroscopy (EIS) was carried out to investigate the charge-transfer kinetics process (Figure 3D). The smallest radius of the 4 wt% Ru-Zn/MWCNTs catalyst demonstrated the lowest charge transfer resistances (R_ct_) and the fastest electron transfer, compared to the 4 wt% Ru/MWCNTs, 4 wt% Zn/MWCNTs and 5 wt% Pt/C catalysts. Therefore, the large electrochemical surface area and efficient charge transfer kinetics endowed the Ru-Zn/MWCNTs with an excellent HER performance in the alkaline conditions, outperforming most of the previously reported Ru-based catalyst (Figure 3E; Supplementary Table S2). Moreover, the HER performance of 4 wt% Ru-Zn/MWCNTs can be maintained for 10,000 cycles of CV sweeps with no obvious change in the LSV curves (Figure 3F). By contrast, the activity of pure 4 wt% Ru/MWCNTs dropped severely, indicating that the incorporation of Zn species can help improve the stability of the Ru-based catalyst. This can also be verified by the chronoamperometric curves (Supplementary Figure S4) that the 4 wt% Ru-Zn/MWCNTs catalyst displayed no observable decay for 90 h of continuous operation, while the current intensity of the 4 wt% Ru/MWCNTs catalyst decreased significantly within only no more than 20 h. Therefore, the addition of Zn species has brought a significant enhancement in both the HER activity and the stability of the Ru-based catalysts.

### 3.5 Analyzing the possible role of Zn species in the Ru-Zn/MWCNTs electrocatalyst

**FIGURE 4:**
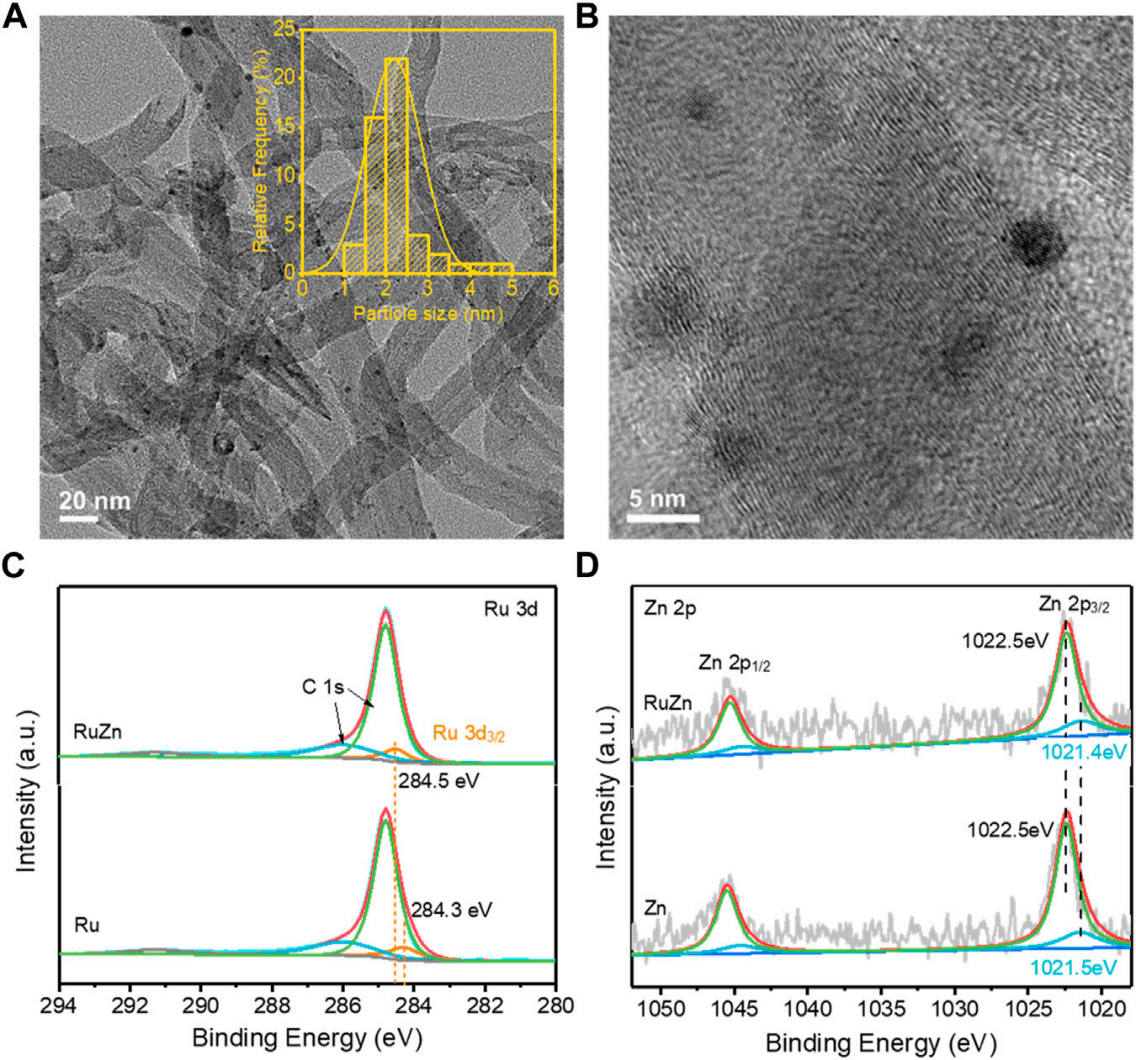
The structural and electronic properties of the 4 wt% Ru-Zn/MWCNTs catalysts and the counterparts. **(A)** TEM images, inset: particle size distribution; **(B)** high resolution TEM images; **(C)** XPS spectra for the Ru 3 days regions of the 4 wt% Ru-Zn/MWCNTs and 4 wt% Ru/MWCNTs samples; **(D)** XPS spectra for the Zn 2p regions of the 4 wt% Ru-Zn/MWCNTs and 4 wt% Zn/MWCNTs samples.

To investigate possible role of Zn species in promoting the activity of Ru/MWCNTs, the structural and electronic properties of the Ru-Zn/MWCNTs catalyst were further characterized. Figure 4A showed the typical TEM images of 4 wt% Ru-Zn/MWCNTs, which demonstrated that monodispersed nanoclusters were homogeneously anchored on the MWCNTs with an average particle size of ca. 2 nm (Figure 4A inset). High-resolution TEM (Figure 4B) also clearly confirmed the existence of nanoclusters on the surface of the MWCNTs. In addition, the ICP analysis showed that the Ru and Zn loadings in the Ru-Zn/MWCNTs catalyst were ca. 2.38 wt% and 1.44 wt%, respectively, which were consistent with the theoretical values. Furthermore, the surface chemical compositions were analyzed by XPS (Figures 4C, D). The Ru 3d peak at binding energy of 284.3 eV was attributed to the Ru 3 d_3/2_ of metallic Ru^0^ in the Ru/MWCTNs sample (Barman, et al., 2017; Yang, et al., 2021; Zhang, et al., 2021). Nevertheless, for the Ru-Zn/MWCNTs sample, the binding energy of the corresponding metallic Ru^0^ peak was positively shifted by about 0.2 eV compared to the pure Ru/MWCTNs, indicating a deficiency in the electrons of the Ru species. In addition, the two peaks at binding energy of 1022.5 eV and 1021.5 eV of the Zn/MWCTNs sample (Figure 4D) can be assigned to Zn 2 p_3/2_ of the Zn^2+^ and Zn^0^ species, respectively, suggesting the coexistence of metallic and oxidized Zn species on the surface (Samanta, et al., 2020; Yan, et al., 2019; Zhu, et al., 2016). However, the Zn 2 p_3/2_ peak corresponding to the Zn^0^ species showed a negative shift to 1021.4 eV for the Ru-Zn/MWCNTs sample, which was in good accordance with the positive changes in Ru 3d peak of the Ru species. This manifested that the addition of Zn species could promote the electron transfer from Ru metals to the Zn species, leading to a strong interaction between Ru and Zn species. Combining with the results of the XRD patterns (Figure 1A), it can be deduced that RuZn alloy may be formed in the Ru-Zn/MWCNTs sample. Therefore, the above results demonstrated that the electronic state of Ru nanoclusters can be modified by addition of Zn species to form RuZn alloy and ZnO. Generally, the formation of Ru-based alloy has been proved to make it easier for the cleavage of the highly-polarized H-OH in water molecules due to the electron donation of Ru to the other metals, thus promoting the process of water adsorption/activation and optimizing the energy barrier of H adsorption/H_2_ desorption (Liu, et al., 2018; Li, et al., 2021). This can explain the promotional effect of Zn species modification on the HER performance brought by formation of RuZn alloy in the Ru-Zn/MWCNTs. On the other hand, it was reported that ZnO was active in adsorbing water at ambient conditions, and hydroxyls were readily to form on its surface (Holdren, et al., 2018). This can explain the important role of ZnO in modulating the OH_ad_ intermediate to promote the water electrolysis. Moreover, the ability of water dissociation can be enhanced by the interface between the metals and metal oxides (Yao, et al., 2013). Hence, the coexistence of RuZn alloy and ZnO species may synergistically regulate the binding energy of H_ad_ and OH_ad_ intermediates in water electrolysis, which may be the main reason for the higher HER activity and stability of Ru-Zn/MWCNTs.

## 4 Conclusion

In conclusion, we presented a highly efficient and stable Ru-Zn/MWCNTs electrocatalyst by incorporating Zn species as modifier into Ru/MWCNTs electrocatalysts. The as-prepared Ru-Zn/MWCNTs electrocatalyst delivered a high HER activity with low overpotentials of 26 and 119 mV at current intensities of 10 and 100 mA cm^−2^, respectively, superior to Ru/MWCNTs at the same Ru loading and the commercial 5 wt% Pt/C catalysts. Moreover, the high activity can be maintained with no obvious decay for a duration test of at least 10,000 cycles and a long-term stability of more than 90 h. The Zn species was found to modify the electronic structures of active Ru species that could modulate the adsorption energy of the H_ad_ and OH_ad_ intermediates, which should be the main reason for the enhanced performance observed. Similar strategy could be extended to the synthesis of various electrocatalysts beyond Ru towards more efficient renewable energy conversion.

## Data Availability

The original contributions presented in the study are included in the article/Supplementary Material, further inquiries can be directed to the corresponding authors.
