# Supplementary material for: Ruthenium nanoclusters modified by zinc species towards enhanced electrochemical hydrogen evolution reaction
Source: Front Chem. 2023 Apr 6;11:1189450. doi: 10.3389/fchem.2023.1189450 (PMC10115985; doi:10.3389/fchem.2023.1189450)
Supplement: Supplementary file 1 [file DataSheet1.docx]

Supplementary Material

Ruthenium nanoclusters modified by zinc species towards enhanced electrochemical hydrogen evolution reaction

Hefeng Zhang^1^, Shengliang Qi^1^, Kaixin Zhu*, Xu Zong*

^1^ These authors contributed equally

*** Correspondence:** Corresponding Author: [xuzong@dlmu.edu.cn](mailto:xuzong@dlmu.edu.cn); kxzhu@dlmu.edu.cn

# Supplementary Figures and Tables

## Supplementary Figures

.
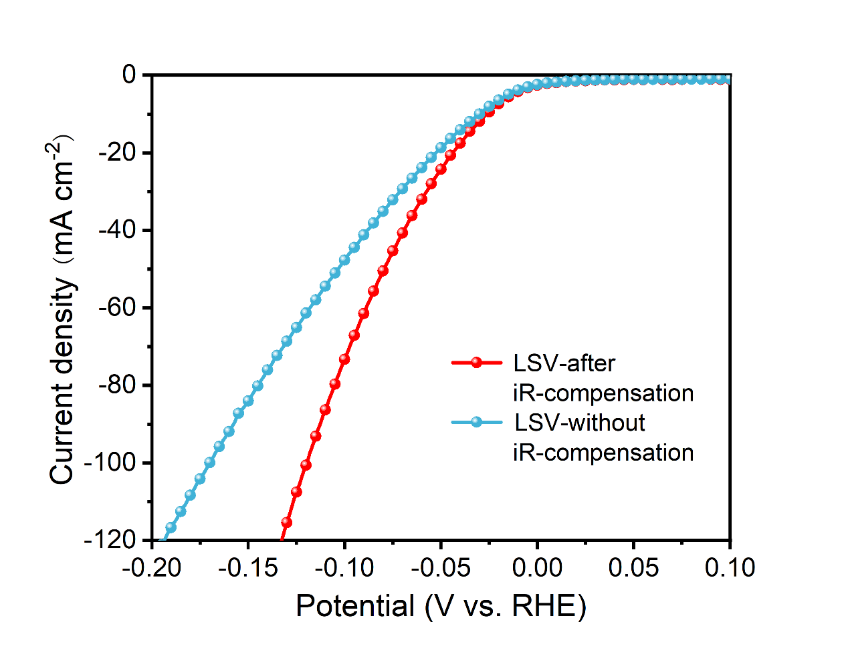


**Supplementary Figure 1.** The LSV curves of 4 wt% Ru-Zn/MWCNTs in 1.0 M KOH with and without iR-compensation.

**Supplementary Figure 2.** Electrochemical HER performances of the Ru-Zn/MWCNTs samples with different amount of Ru and Zn contents.


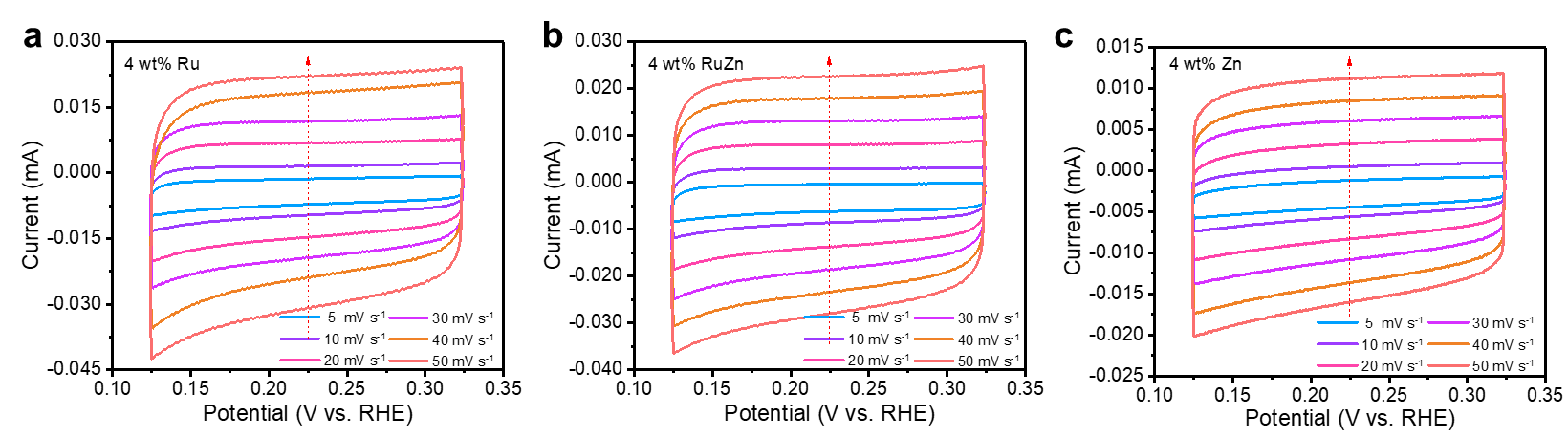


**Supplementary Figure 3.** Cyclic voltammograms (CVs) at different scan rates of the different catalysts to evaluate the electrochemical active surface area (ECSA). (a) 4 wt% Ru/MWCNTs; (b) 4 wt% Ru-Zn/MWCNTs; (c) 4 wt% Zn/MWCNTs.

The electrochemical surface areas (ECSA) were acquired from CV curves at the potential window ranging from 0.124 to 0.324 V vs. RHE at different scan rates of 5, 10, 20, 30, 40, 50 mV s^-1^, respectively. By plotting the Δj/2_0.224 V vs. RHE_ against scan rate curves, the linear slopes were obtained, which were further divided by the specific capacitance (40 μF cm^-2^) to obtain the ECSA of each sample. Then, the LSV curves of specific activity were obtained from the current normalized by ECSA.

**Supplementary Figure 4.** Cyclic voltammograms (CVs) at different scan rates of the different catalysts to evaluate the electrochemical active surface area (ECSA). (a) 4 wt% Ru/MWCNTs; (b) 4 wt% Ru-Zn/MWCNTs; (c) 4 wt% Zn/MWCNTs.

## Supplementary Tables

**Supplementary Table 1.** The HER performance comparison of the different Ru-based catalysts in our study.

| **Catalysts** | **Overpotential (mV)**  **10 mA cm^-2^** | **Overpotential (mV)**  **100 mA cm^-2^** | **Tafel slopes**  **(mV dec^-1^)** |
| --- | --- | --- | --- |
| 4 wt% Ru-600 | 24 | 122 | 46.7 |
| 2 wt%Ru-Zn (1:1) | 53 | 191 | 54.2 |
| 4 wt% Ru-Zn (1:1) | 26 | 119 | 44.5 |
| 8 wt% Ru-Zn (1:1) | 22 | 114 | 34.1 |
| 16 wt% Ru-Zn (1:1) | 16 | 82 | 27.8 |
| 4 wt% Ru-Zn (1:2) | 39 | 143 | 52.2 |
| 4 wt% Ru-Zn (2:1) | 26 | 127 | 39.8 |

**Supplementary Table 2.** Comparison of the representative Ru-based HER catalysts reported previously.

| Catalyst | Overpotential @10 mAcm^-2^ | Tafel slope  (mV dec^-1^) | Reference |
| --- | --- | --- | --- |
| 4 wt% Ru-Zn | 26 | 44.5 | This work |
| 16 wt% Ru-Zn | 16 | 27.9 | This work |
| S-RuP@NPSC-900 | 92 | 90.2 | Adv. Sci. 2020, 7, 2001526. |
| ah-RuO_2_@C | 63 | 62 | Nano Energy 2019, 55, 49. |
| RuP_2_@NPC | 52 | 69 | Angew. Chem. 2017, 56, 11559. |
| Ru@CN | 32 | 53 | Energy Environ. Sci. 2018, 11, 800. |
| Ru/C_3_N_4_/C | 79 | 49 | J. Am. Chem. Soc. 2016, 138, 16174. |
| CoRu@NC | 32 | 47 | Nanotechnology 2018, 29, 225403. |
| [0.27-RuO](mailto:0.27-RuO2@C)_[2](mailto:0.27-RuO2@C)_[@C](mailto:0.27-RuO2@C) | 33 | 53 | Nano Energy 2019, 55, 49-58. |
| Ru-GLC | 35 | 46 | ACS Appl. Mater. Interfaces 2016, 8, 35132-35137. |
| Ru^0^/TiO_2_ | 41 | 52 | J. Colloid Interface Sci. 2018, 531, 570-577 |
| Ru/CeO_2_ | 47 | 41 | ACS Appl. Mater. Interfaces 2018, 10, 6299-6308. |
| RuP_x_@NPC | 51 | 46 | ChemSusChem 2018, 11, 743-752 |
| Ru-MoO_2_ | 55 | 44 | J. Mater. Chem. A 2017, 5, 5475-5485. |
| Ru/HMCs-500 | 26.9 | 45.7 | J. Mater. Chem. A 2023, 11, 3524-3534. |
| MoP-Ru_2_P/NPC | 47 | 36.9 | Appl. Catal. B, 2022, 303, 120879. |
| Ru MNSs | 24 | 38 | Angew. Chem. Int. Ed. 2022, 61, e202116867. |
| Ru-NiCo_2_S_4_ | 32 | 41.3 | Adv. Funct. Mater. 2022, 32, 2109731 |
| V_O_-Ru/HfO_2_-OP | 39 | 29 | Nat. Commun. 2022, 13, 1270. |
| Ru-Ni_0.85_Co_0.15_Se/NF | 18.2 | 35.6 | Applied Catalysis B: Environmental 2023, 327, 122466. |
